# Alcohol Consumption Modulates the Development of Chronic Pain in COVID-19 Patients: A Network Meta-Analysis

**DOI:** 10.1021/acsptsci.4c00479

**Published:** 2025-02-04

**Authors:** Muhammed Bishir, Michael Vigorito, Ming-Huan Chan, Mohammed A S Khan, Sulie L. Chang

**Affiliations:** 1 Institute of NeuroImmune Pharmacology, Seton Hall University, South Orange, New Jersey 07079, United States; 2 Department of Biological Science, Seton Hall University, South Orange, New Jersey 07079, United States; 3 Institute of Neuroscience, National Chengchi University, Taipei 116,Taiwan; 4 Department of Medical Research, China Medical University Hospital, Taichung 40447, Taiwan; 5 Department of Neurosurgery, Brigham & Women’s Hospital, Boston, Massachusetts 02115, United States

**Keywords:** long COVID, alcohol-induced pain, QIAGEN knowledge base, ingenuity pathway analysis, SARS-CoV-2 infection

## Abstract

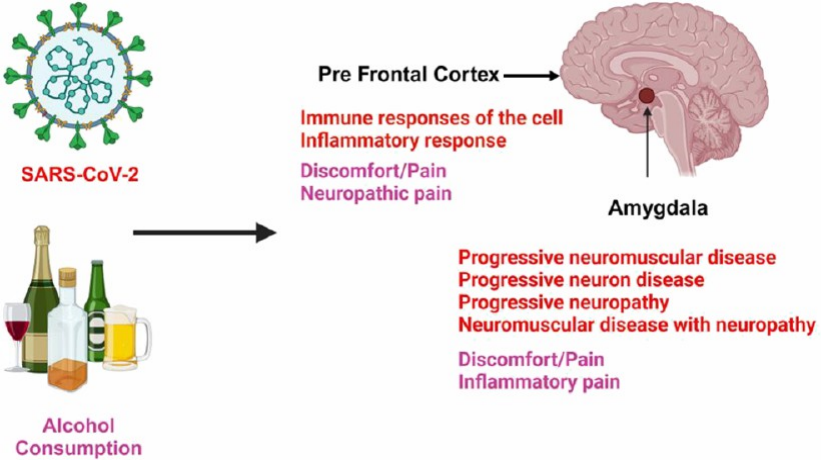

The mechanisms underlying the onset and progression of chronic pain in COVID-19 patients have been understudied. Using network meta-analysis, we previously demonstrated that alcohol augments COVID-19 symptoms and pathologies possibly by inducing a severe cytokine storm. We and others have also reported that acute alcohol consumption produces analgesic effects, while chronic alcohol consumption results in hyperalgesia and chronic pain. This study aimed to identify the influence of alcohol consumption and COVID-19 on pain. Using publicly available curated gene expression data sets of differentially expressed genes (DEGs) in the prefrontal cortex (PFC) and amygdala of COVID-19 patients, we employed a bioinformatics application, QIAGEN ingenuity pathway analysis (IPA), to identify the key signaling pathways, upstream regulators, and biological functions in these brain areas known to play a role in pain. Canonical pathway analysis revealed activation of the neuropathic pain pathway and signaling pathways involving the cytokine storm, S100 family, IL-6, and neuroinflammation. IPA’s network builder was employed to construct a network map of shared molecules between alcohol and pain-related constructs (discomfort, neuropathic pain, and inflammatory pain). The simulation of alcohol consumption inhibited pain in this network map. To study the influence of COVID-19, we overlaid the DEGs from the PFC and amygdala onto these networks, mimicking alcohol consumption during SARS-CoV-2 infection. Upregulation of molecules in the amygdala and PFC predicted an increase in neuropathic pain, as well as an increase in inflammatory pain in the PFC. Our results suggest that while alcohol consumption directly inhibits pain, the presence of COVID-19 exaggerates impaired cytokine signaling, neuroinflammation, and neuropathic pain signaling in the CNS providing novel insights into the signaling pathways associated with chronic pain of the COVID-19 patients.

COVID-19 disease is caused by the severe acute respiratory syndrome coronavirus-2 (SARS-CoV-2), which was declared a global pandemic by the World Health Association (WHO) March 11, 2020, accounting for over 775 million cases and 7 million deaths across the world.^1^ In addition to triggering pneumonia that can progress to acute respiratory distress syndrome and impact the cardiovascular system,^2^ the original SARS-CoV-2 virus (Wuhan) and the subsequent variants of concern (e.g., Gamma, Delta & Omicron) can also gradually invade the central nervous system (CNS) directly through the blood (hematogenous spread) and by axonal transport in olfactory sensory neurons or indirectly by neurovirulence pathologies such as peripheral cytokine storm, damaged blood–brain barrier and immune activation.^3−5^ COVID-19 associated with CNS symptoms include loss of taste and smell, headache, dizziness, and pain.^6,7^ However, the long-term effects (long COVID) include cognitive impairment and neurodegenerative conditions.^8,9^ Neuropathic pain is a symptom that can occur in the acute and long phases of COVID-19 phases. Clinical case reports from COVID-19 patients described the onset of neuropathic pain in COVID-19 patients from 3 to 45 days post-SARS-CoV-2 infection.^10^ A meta-analysis found a greater prevalence of neuropathic pain in long COVID compared to the acute phase of the disease.^5^

It must be noted, however, that pain terms and definitions are not consistently defined in the literature and also undergo changes as research advances, especially as it is applied in the clinical setting. *The International Association for the Study of Pain*, for example, recently revised its definition of neuropathic pain as a clinical description of the perception of pain resulting from the diseased state of the somatosensory nervous system serving no protective function, in contrast to the protective function of nociceptive pain which is induced by a noxious stimulus indicating actual or potential tissue damage^11^. The term “discomfort” is also used in the literature but in a manner that is (1) not distinguished from pain, (2) considered as a concept that is separate but overlapping with pain, or (3) described as resulting from pain.^12^ What makes it difficult to clearly distinguish discomfort from chronic pain in particular is that both concepts involve physical and psychological components, the latter dependent primarily on the subjective reports of patients.^13^ Apart from discomfort and neuropathic pain, COVID-19 patients also reported various types of pain such as joint contractures, headache, muscle atrophy, or pain due to critical illness myopathy or polyneuropathy.^14^ Pain in COVID-19 patients is attributed to chronic inflammation induced by the SARS-CoV-2 infection. Inflammatory pain is an acute protective response associated with tissue damage that may motivate recuperative behavior to prevent reinjury, but conditions of chronic inflammation can lead to chronic pain. Viral infection triggers neuropathy which involves immune-related mechanisms as indicated by several viral genomic studies of neuropathy.^15,16^ Risk factors that aggravate and potentially worsen the pain conditions in the COVID-19 pathologies include cytokine storm, compromised immune system, and neuroinflammation.

Alcohol misuse and alcohol use disorder (AUD) are considered risk factors, as they exacerbate viral lung infection, cytokine storm, and pneumonia.^17^ We have previously reported that alcohol consumption augmented COVID-19 pathology by activating TNF, and inflammatory signaling pathways including IL1, IL2, IL6, and HMGB1 signaling pathways.^18^ Combined effects of alcohol and infection by the SARS-CoV-2 on cytokine storm and inflammatory signaling pathways have been shown to be mediated by the activation of NLRP3 inflammasome.^17,19^ The pronounced inflammation in response to SARS-CoV-2, coupled with alcohol misuse, can further lead to neuroinflammation and exacerbate pain symptoms in the CNS of COVID-19 patients. There are no studies that have been reported specifically addressing pain in the context of COVID-19 and alcohol misuse. In previous centuries, alcohol was believed to have analgesic properties and was even used during medical procedures^20^ to reduce pain intensity and increase pain threshold.^21,22^ However, recent findings have reported that chronic exposure results in mechanical allodynia and neuropathic pain^23^ (for review see^13^). Hence, understanding the combined effects of alcohol and infection by SARS-CoV-2 on various pain conditions is crucial.

Thus, we hypothesize that alcohol misuse among COVID-19 patients could increase the incidence of pain and escalate pain-related conditions after infection by the SARS-CoV-2. Major disadvantages in COVID-19 research include limited access to COVID-19 tissue samples. Additionally, no rodent models could be generated with the SARS-CoV-2 infection to mimic the infection, and there are ethical challenges in clinical research. Therefore, utilizing bioinformatics methodology, equipped with vast database archives of biological data that are archived in the databases to analyze transcriptomic data, is a useful tool in understanding complex pathological conditions. Here, we employed QIAGEN knowledge base (QKB) and ingenuity pathways analysis (IPA) to identify the molecules related to ethanol (EtOH), SARS-CoV-2, and various pain conditions. We also utilized transcriptomic data from the National Center for Biotechnology Information (NCBI) Gene Expression Omnibus (GEO) database-GSE188847, which encompasses gene expression changes in the prefrontal cortex (PFC) of COVID-19 patients. Transcriptomic data from the amygdala of COVID-19 patients were performed by Piras et al.^24^ Differentially expressed genes (DEGs) obtained from both PFC and amygdala have been analyzed, employing QKB-IPA to study the combined effect of alcohol misuse and SARS-CoV-2 on various pain conditions. Pain signals from the spinal cord reach the brain, where they are processed in various brain regions. Previous reports show that several of the brain regions that are involved in processing pain signaling overlap with brain regions involved in alcohol-induced intoxication, withdrawal, and anticipation.^25−27^ From these studies, we found that four brain regions, namely, the PFC, nucleus accumbens (NAc), central amygdala (CeA), and ventral tegmental area (VTA), are central to alcohol intoxication, withdrawal, anticipation, and chronic pain. PFC is involved in higher-order pain signal processing and regulates cognitive components of pain. It also interacts with other brain regions, including the amygdala, thalamus, and nucleus accumbens, which are all part of the central pain matrix. The amygdala is mainly involved in processing the emotional components of pain.^28,29^ In the context of COVID-19, both imaging and transcriptomic studies report significant alterations of the PFC and amygdala in COVID-19 patients.^24,30,31^ SARS-CoV-2 mediated neuroinflammation may result in the impaired functioning of the PFC and amygdala, resulting in impaired cognitive and emotional responses and increased pain perception. With these premises, we selected PFC and amygdala to study how alcohol exposure during COVID-19 contributes to heightened pain responses. The present study showed that the simulation of EtOH, mimicking alcohol exposure, inhibits pain signaling, whereas activating commonly shared molecules between EtOH and COVID-19 increases the pain. We also found that the activation of inflammatory responses in PFC, neuropathy in the amygdala, and EtOH simulation of DEGs from both regions increased pain responses.

## 2 Material and Methods

### 2.1 QIA2GEN Knowledge Base (QKB)

The QKB is a comprehensive repository of biological information that fuels QIAGEN-IPA. These are composed of over seven million individually modeled relationships between diseases, drugs, biological entities, (*e.g.,* genes, proteins, metabolites), and processes (*e.g.,* expression, molecular cleavage, and phosphorylation). The content of the QKB can be accessed via IPA software. The annual license for IPA software was purchased from QIAGEN (Germantown, MD). The data in this study were collected from the QKB, between August and November 2023, for bioinformatics analysis.

### 2.2 Ingenuity Pathway Analysis

IPA has comprehensive information about molecular interactions and relationships for biological understanding of large data sets, including Transcriptomics, miRNA expression, proteomics, phosphoproteomics, genomics, and metabolomics. This bioinformatics application generates data, performs background statistics, analyzes the data with the seven million curated findings in the QKB, and outputs the canonical pathways, upstream regulators, and diseases and functions associated with the data set. We used IPA’s “grow” tool to identify the canonical pathways and molecules associated with EtOH, the SARS-CoV-2, and various pain conditions (nodes), including discomfort, neuropathic pain, and inflammatory pain. The “Compare” tool was used to identify the commonly shared molecules between two nodes, and the “Connect” tool was then used to draw networks between EtOH and downstream molecules and disease nodes based on the research findings in the QKB. Thus, these tools allow for a more holistic view of biological processes by incorporating multiple pathways and the known interactions between their component molecules. IPA’s molecular activity predictor (MAP) tool was then utilized to predict the upstream and/or downstream effects of activation or inhibition of molecules in a network or pathway given one or more neighboring molecules with “known” activity. The projected relationships between molecules on the network maps are indicated with color-coded connecting lines: orange indicates activation, blue indicates inhibition, gray indicates no available prediction, and yellow indicates findings that are inconsistent with downstream molecules.

**Figure 1:**
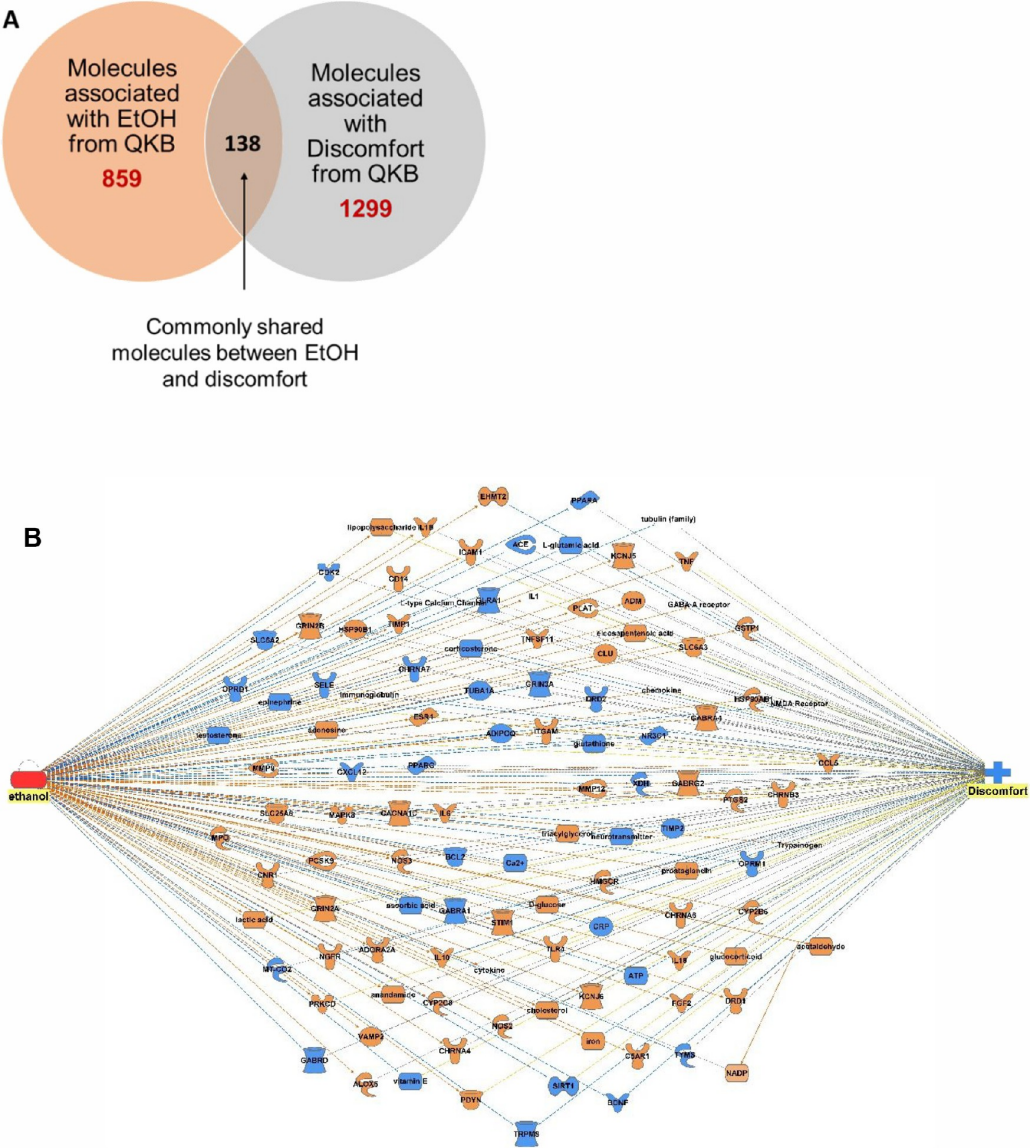
Impact of alcohol exposure on discomfort. (A) Venn diagram showing 138 molecules, which are commonly shared between EtOH and discomfort. (B) EtOH simulation of activation of the overlapping molecules between EtOH and discomfort that participate in the inhibition of discomfort or pain. Orange connecting lines indicate activation, blue indicate inhibition, and gray indicate that the z-score is zero and no prediction can be made.

We also report the results of a simulation of the effects of alcohol misuse within the context of the relevant biological system to identify the effects of EtOH on downstream molecules and functions. The statistical significance of EtOH-induced inhibition or enhancement of discomfort (Figure 1B) was calculated using the mathematical formula created by Krämer et al.^32^ and as employed and described in our previous publications.^18,33−36^ The formula calculates a local z-score for each molecule based on the sum products of the findings in the literature that support the observed relationship. Then, an overall z-score, ranging from −2 to +2, is calculated from the local z-score for the EtOH-induced inhibition or enhancement of discomfort; a negative z-score strongly supports an inhibitory relationship, while a positive z-score strongly supports an activation relationship.

### 2.3 Transcriptomic Data Set of PFC and Amygdala From COVID-19 Patients

The GEO (https://www.ncbi.nlm.nih.gov/geo/) is an international public repository for microarray and next-generation sequence functional genomic data sets submitted by researchers. The resource supports archiving of raw data, processed data, and metadata, which are indexed, cross-linked, and searchable. All data are freely available for download in a variety of formats. In the present study, we used the data set GSE188847 (https://www.ncbi.nlm.nih.gov/geo/query/acc.cgi?acc=GSE188847), which encompasses Gene expression changes in the PFC of COVID-19 patients. The data set included 22 COVID-19 patients (male n = 12; female n = 10) aged between 23 and 84 and uninfected age and sex-matched controls. All the samples were confirmed positive for SARS-CoV-2 by nasopharyngeal swab qPCR either premortem or perimortem. The subjects did not have any history of neurological disorders, with two individuals having a history of stroke.^30,37^ The data set contains gene expression data from PFC samples of COVID-19 patients and control samples. GEO2R was used to identify the DEGs in the PFC in response to SARS-CoV-2 infection. The DEGs were uploaded to IPA for further enrichment analysis.

DEGs in the amygdala of COVID-19 patients were obtained from a preprint from Piras et al.^24^ The data set included 20 COVID-19 patients (11 males and 9 females) aged between 30 and 90 and 20 uninfected non-COVID-19 patients serving as the control group. All COVID-19 subjects were confirmed positive for SARS-CoV-2 infection and died from severe COVID-19. The list of DEGs in the amygdala of COVID-19 was made available in the supplementary file by Piras et al.^24^ The DEGs were analyzed using a QIAGEN IPA to identify the signaling pathways.

### 2.4 IPA Core Analysis-Signaling Pathways, Upstream Regulator, and Regulator Effects

For statistical significance, IPA calculates the probability of an association of DEGs in the uploaded data sets with canonical pathways in the QKB. IPA uses the right-tailed Fisher’s exact test to determine if there is a significant over-representation (enrichment) in the data set of molecules associated with a canonical pathway, compared with what you would expect by chance. The test considers the number of genes in the uploaded DEG data set, the reference set of genes (canonical pathways) from QKB, and the number of genes that overlap between the two organized as a 2 × 2 contingency table. The calculated *p*-value suggests that the genes are either significantly enriched in the data set (*p* < 0.05) or that any association is likely due to chance (*p* > 0.05).

The “regulatory effect analysis” identifies the potential pathways among the upstream regulatory networks and downstream functions associated with the identified DEGs. IPA constructs directional visualized networks using the uploaded DEG data set and the wide range of curated literature in the QKB to generate causal hypotheses. Using the uploaded DEG data set as intermediary molecules a network merges the known signals from upstream regulators to downstream outcomes including functions and diseases.^32,34,38^ As seen in figures (Figures 4 and 5) a network has a hierarchical structure with upstream regulation to the left (or on top), the data set DEGs in the middle, and the downstream biological functions and diseases to the right (or at the bottom). Connections between upstream regulators and downstream functions and diseases establish causal hypotheses. Any upstream regulator connected to a data set DEG but not connected with the downstream function or disease may suggest a possible novel association not yet described in the literature. For each network constructed, IPA computes a consistency score, which is a measurement used to rank the regulator networks of interest. The consistency score measures the causal consistency and intensity of the connectivity between upstream regulators, DEGs in the data set, and diseases and functions with what would be expected from the findings in the QKB; a higher consistency score determines the most accurate regulatory effect. The consistency score itself is not a measure of statistical significance but a rule of thumb to rank a set of statistically significant networks within the same core analysis settings. The formula to calculate the consistency score is
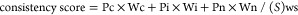


Pc: number of consistent paths from regulator to downstream functions via genes in the data set.

Wc: weight that rewards for consistent path (1, for a consistent path).

Pi: total number of inconsistent paths.

Wi: weight that penalizes inconsistent paths (set to −1.5).

Pn: total number of noncausal paths.

Wn: weight for noncausal paths (set to 0).

*S*: total number of data set targets.

Ws: penalty weight for the size of the network (set to 0.5).

“Comparison analysis” enables visualization across multiple analyses like signaling pathways, upstream regulators, and diseases and functions to understand the enrichment and activation states of pathways among the data sets. The comparison analysis was used to compare the core analysis results of the DEGs from the amygdala and PFC of COVID-19 patients.

## 3 Results

### 3.1 Exposure to Alcohol Promotes Analgesia

A total of 997 molecules associated with EtOH and 1437 molecules associated with discomfort were obtained from QKB. Figure 1 shows the association between EtOH and discomfort. The Venn diagram shows 138 molecules overlapping between EtOH and discomfort (Figure 1A). A network map depicting the biological relationships between EtOH, 138 overlapping molecules, and discomfort was constructed. Molecular activity predictor (MAP) analysis of the EtOH-simulation activation mimicking alcohol consumption inhibited the discomfort (Figure 1B). The z-score EtOH-mediated inhibition of discomfort was found to be −1.46, suggesting inhibition of discomfort.

### 3.2 Molecules Commonly Shared between EtOH and COVID-19 Elevate Discomfort

**Figure 2:**
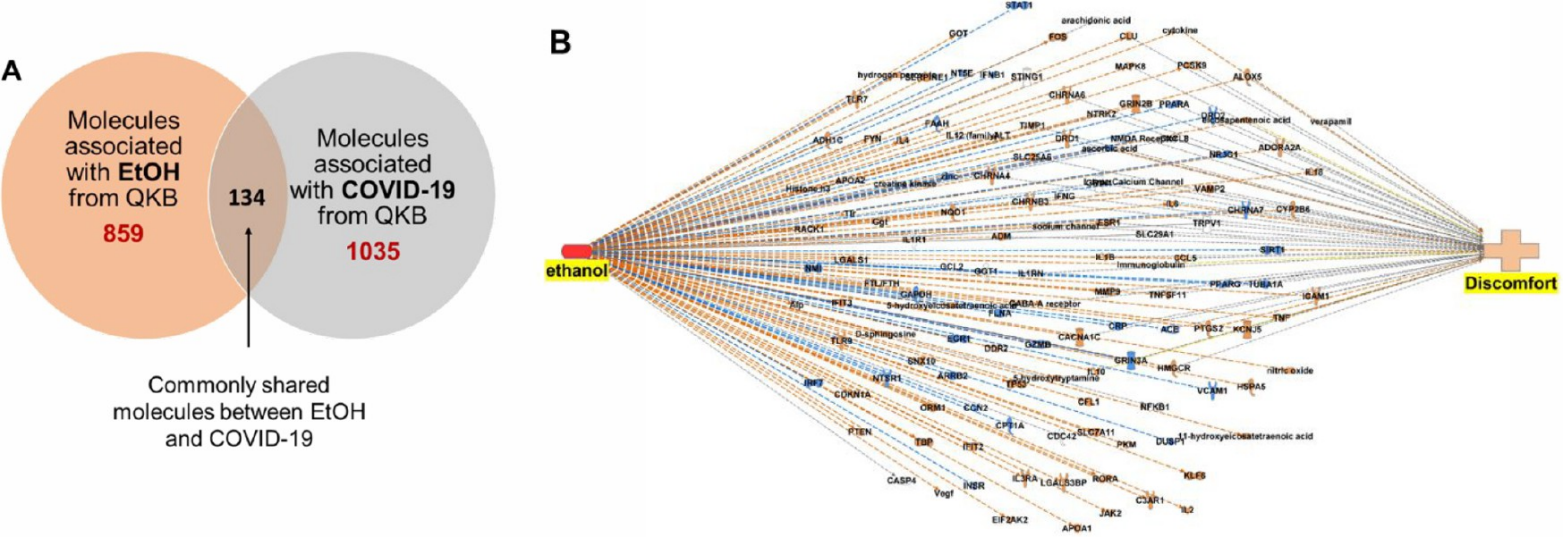
Impact of alcohol and SARS-CoV-2 infection on discomfort. (A) Venn diagram showing 134 molecules were found to be commonly shared between EtOH (859 molecules) and SARS-CoV-2 infection (1035 molecules). (B) EtOH simulation activation of the overlapping molecules between EtOH and COVID-19 resulted in increased discomfort. Orange connecting lines indicate activation, blue indicate inhibition, and gray indicate that the z-score is zero and no prediction can be made.

A total of 134 molecules were commonly shared between 859 EtOH- and 1035 COVID-19 infection-associated molecules (Figure 2A). IPA’s “path explorer” constructed a network map based on the relationship between the shared molecules of EtOH and COVID-19. Increased levels of EtOH simulation (EtOH simulation activation), mimicking alcohol misuse, led to the concurrent activation and inhibition of the overlapping molecules, resulting in increased SARS-CoV-2 infection. Increased levels of EtOH-mediated elevation of cytokines, IL-6, PTGS-2, and TNF; and inhibition of SIRT-1 and ascorbic contributed to the increased discomfort. Eicosapentoinic acid, immunoglobulin, and IL10 were also elevated following EtOH simulation activation; however, the impact of these molecules on discomfort was inconsistent in the existing literature. EtOH-mediated activation of ADM, ADORA2A, ALOX5, CACNA1C, CCL5, CHRNA4, CHRNA6, CHRNB3, CLU, CYP2B6, DRD1, ESR1, GABA-A receptor, GRIN2B, HMGCR, ICAM1, IL18, IL1B, KCNJ5, L-type calcium channel, MAPK8, MMP9, PCSK9, SLC25A6, TIMP1, TNFSF11, and VAMP2 and inhibition of ACE, CHRNA7, CRP, DRD2, GRIN3A, NMDA receptor, NR3C1, PPARA, PPARG, and TUBA1A did not show any simulation changes onto discomfort (Figure 2B). The z-score for the EtOH-mediated increase in discomfort was found to be 1.37, a positive z-score, suggesting an increase in discomfort.

### 3.3 Identification and Core Analysis of the Differentially Expressed Genes in the Prefrontal Cortex and Amygdala of COVID-19 Patients

**Figure 3:**
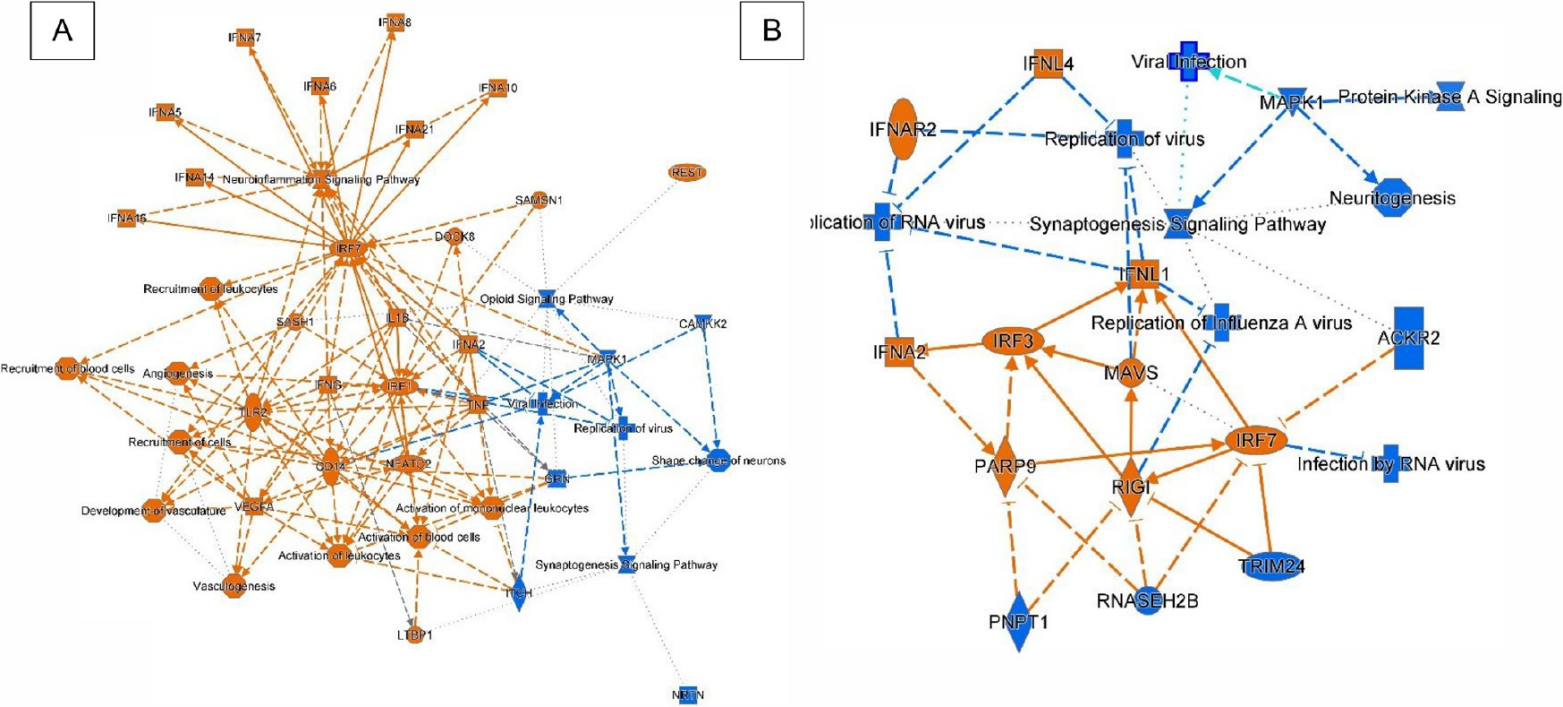
Summary of the core analysis: the figure shows the top signaling pathways and functions and the upstream regulators that mediate the observed activation (orange) or inhibition (blue) of the signaling pathways and functions in the (A) PFC, (B) Amygdala of COVID-19 patients.

DEG analysis of the PFC of COVID-19 patients revealed 8722 significant DEGs (*p*-value < 0.05) compared to control patients (the list of DEGs is provided in the Supporting Information File 1). In the amygdala of COVID-19 patients, there were 1278 significant DEGs (*p*-value < 0.05) compared to controls (the list of DEGs is provided in the Supporting Information File 2). IPA’s core analysis of the DEGs identified the enriched canonical signaling pathways and upstream regulators in COVID-19 patients. The graphical summary provides an overview of the top enriched signaling pathways and functions and the upstream regulators that mediate the activation and inhibition of the signaling pathways and upstream regulators. The pathways activated in the PFC were neuroinflammation, inhibition of opioid signaling, and synaptogenesis signaling pathways. Changes were observed in functions such as the activation of blood cells, leukocytes, and mononuclear leukocytes, as well as the development of vasculature and the recruitment of blood cells and of leukocytes. Vasculogenesis was activated but functions like viral infection and replication of the virus as well as change in shape of neurons were inhibited in the PFC of COVID-19 patients. The observed activation or inhibition of the signaling pathways and upstream regulators were mediated through the activation of upstream regulators including CD14, DOCK8, IFNA2, IFNA5, IFNA6, IFNA7, IFNA8, IFNA10, IFNA14, IFNA16, IFNA21, IFNG, IL1B, IRF1, IRF7, LTBP1, NFATC2, REST, SAMSN1, SASH1 TLR2, TNF, and VEGFA and inhibition of ITCH, GRN, NRTN, MAPK1, and CAMKK2 (Figure 3A). In the amygdala, core analysis of the DEGs showed inhibition of synaptogenesis and protein kinase A signaling pathways. Functions like infection by RNA virus replication of RNA virus and neurogenesis were also found to be inhibited in the amygdala of COVID-19 patients. Inhibition of the signaling pathways and functions were mediated via the upstream regulators including IFNA2, IFNAR2, IFNL1, IFNL4, IRF3, IRF7, MAVS, PARP9, and RIGI (activated) and ACKR2, PNPT1, RNASEH2B, TRIM24, and MAPK1 (inhibited).

### 3.4 Top Upstream Regulator IFNAR Family, Modulates Inflammatory and Immune Response in the PFC of COVID-19 Patients

**Figure 4:**
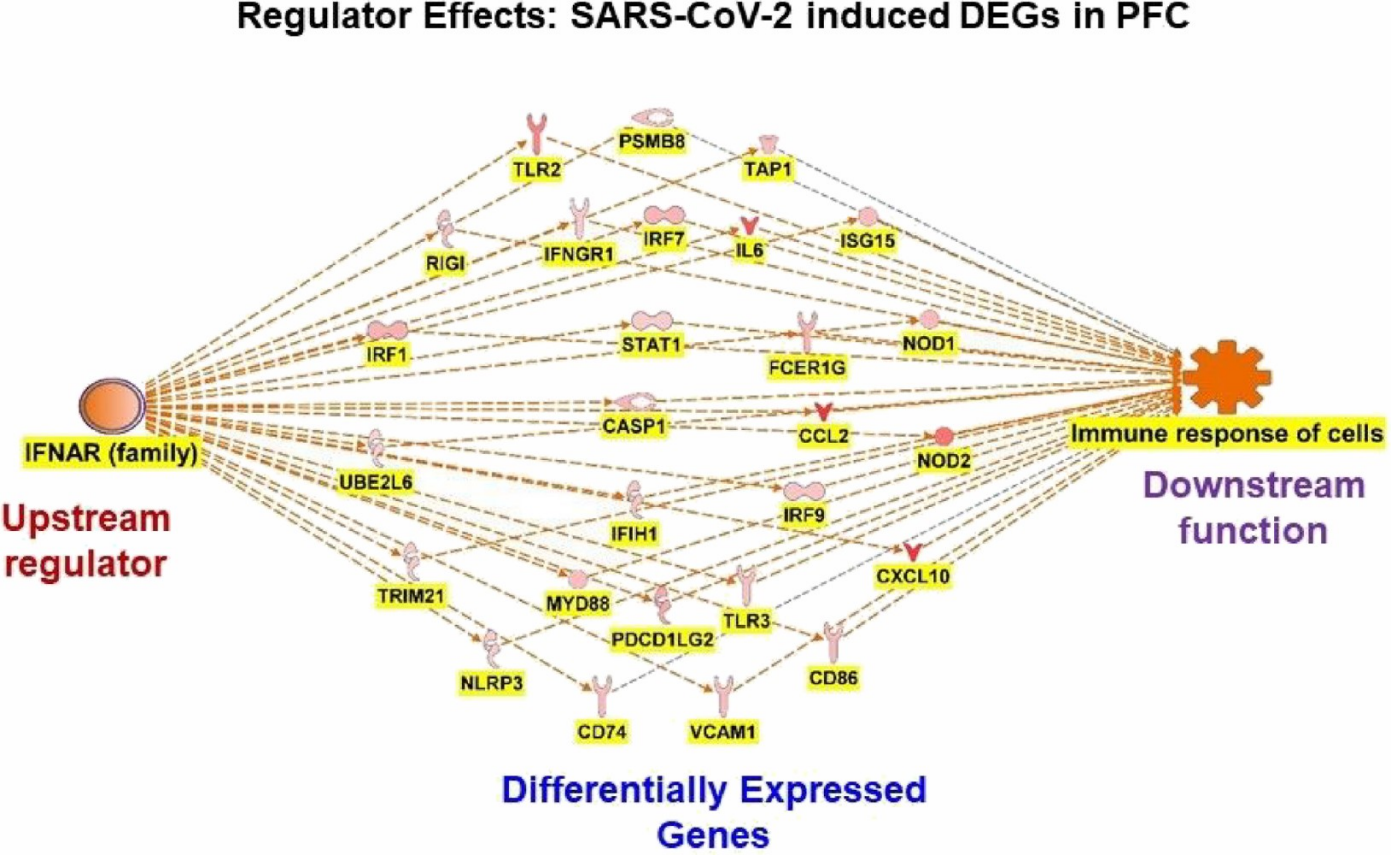
SARS-CoV-2 infection triggers upstream regulator IFNAR results in the activation of the DEGs in the data set and modulates the activation of immune response of cells in the PFC.

Regulatory effects of the upstream regulators in the PFC of COVID-19 patients identified 1864 possible interaction networks between upstream regulators, DEGs in the data set, and the functions they regulate. The top regulatory effect, with a consistency score of 4.619, suggests that the upstream regulator IFNAR mediates the activation of the immune responses of cells. IFNAR-induced activation of immune response of cells was mediated via the activation of DEGs- CASP1, CCL2, CD74, CD86, CXCL10, FCER1G, IFIH1, IFNGR1, IL6, IRF1, IRF7, IRF9, ISG15, MYD88, NLRP3, NOD1, NOD2, PDCD1LG2, PSMB8, RIGI, STAT1, TAP1, TLR2, TLR3, TRIM21, UBE2L6, and VCAM1 (Figure 4).

### 3.5 Upstream Regulators of SARS-CoV-2 Infection-induced DEGs in the Amygdala Modulate Neurological Diseases through the DEGs

Regulatory effects of the DEGs in the amygdala of COVID-19 patients identified 31 combinations of interaction between upstream regulators, DEGs, and associated diseases or functions. The top regulator effect, with the highest consistency score of 60.75, revealed that the combined activation or inhibition of the upstream regulators in the network including IFN, FCGR2A, NONO, NVAS, IFNl1, STAT2, STING1, RNY3, IFNA1, RIPK2, and FCGR2A (activated) and RNASEH2B, TREX1, Ttc39aos1, Irgm1, USP18, ACKR2, IRGM, and RXRB (inhibited) resulted in the increased measurement of progressive neuromuscular and motor neuron diseases as well as progressive neuropathy and neuromuscular disease with neuropathy. The signal for the increased measurement of progressive neuromuscular disease was mediated through the DEGs from the data set including OAS3, ISG15, MX1, EPSTI1, and IRF7. Progressive motor neuron disease was mediated via the activation of DEGs; CSF1, EPSTI1, GSDMD, IFI35, IRF7, ISG15, MX1, OAS3, PSMB8, RNF213, and TRIM5 and the inhibition of APP and IGF1. Progressive neuropathy and neuromuscular diseases with neuropathy were mediated through the activation of CSF1, EPSTI1, GSDMD, IFI35, IRF7, ISG15, MX1, OAS3, PSMB8, RNF213, TRIM5, and C4A and the inhibition of APP and IGF1. Upstream regulators RNASEH2B, TREX1, and RXRB (inhibited) and MVAS and IFN (activated) regulate the inhibition of shape change of neurites, and this inhibition was mediated via the inhibition of APP, IGF1, and DNM3, and the activation of C4A and RELB.

### 3.6 Influence of EtOH and SARS-CoV-2 Infection-induced DEGs in the PFC on Pain Nodes

To identify the impact of alcohol misuse on pain during COVID-19, we performed a simulation analysis of EtOH on DEGs and pain nodes. There were 548 commonly shared molecules between EtOH and DEGs from the PFC of the COVID-19 patients (Figure 6A). IPA’s “path explorer” depicted a network map between EtOH, commonly shared DEGs, and three pain nodes- discomfort, neuropathic pain, and inflammatory pain. Enhanced EtOH in the simulation, mimicking alcohol misuse, increased discomfort, or reversed alcohol’s analgesic properties. Enhanced EtOH in the simulation activated the expressions of C5AR1, IL6, KCNJ6, PDYN, PRKCD, PTGS2, TLR4, and TNF and inhibited the expression of SIRT1; these concurrent activations and inhibitions resulted in increased discomfort. EtOH-mediated activation of EHMT2, NGFR, and IL-10 and inhibition of BDNF and TRPM8 did not contribute to increased discomfort as the literature findings on the effect of these molecules were inconsistent with the state of downstream effect (noted in yellow line). The remaining molecules showed simulation changes following EtOH exposure; however, their effect on discomfort was unpredicted due to limited information in the literature (noted in gray line) (Figure 6B). Neuropathic pain was increased following EtOH exposure, and EtOH simulation-mediated activation of TLR4 in turn resulted in the increased measurement of neuropathic pain (Figure 6C). Enhanced EtOH in the simulation did not show any change in the activity of inflammatory pain, likely due to the contrasting findings in the literature (Figure 6D).

### 3.7 Influence of EtOH and SARS-CoV-2 Infection-induced DEGs in the Amygdala on Pain Nodes

In Figure 7, it is shown that 39 molecules were commonly shared between EtOH and DEGs from the amygdala of COVID-19 patients (Figure 7A). IPA’s “path explorer” depicted a network map between EtOH, commonly shared DEGs, and three pain nodes: discomfort, neuropathic pain, and inflammatory pain. Simulation of EtOH, mimicking alcohol misuse, resulted in an increased level of measurement of discomfort. Enhanced EtOH activated KCNJ6, which, in turn, resulted in increased discomfort. Molecules like NOS3, GABRG2, GRIN2B, and HSP90AB1 were activated following EtOH simulation activation; however, their effect on discomfort was unpredicted due to limited information in the literature (noted in gray line) (Figure 7B). Among the 39 commonly shared molecules, there were no molecules associated with inflammatory pain, and IPA’s path explorer tool predicted a set of downstream molecules from the DEGs that could affect inflammatory pain. Enhancement of EtOH in simulation in this network resulted in the increased measurement of inflammatory pain (Figure 7C). Among the overlapping DEGs, only one molecule of CACNA2D1 was associated with neuropathic pain. Similar exposure to EtOH did not show any simulation change in the expression of CACNA2D1 as well as neuropathic pain (Figure 7D).

## 4 Discussion

In recent years, the usage of computational tools in biological research has advanced our understanding of the pathology of the COVID-19 pandemic, which is caused by SARS-CoV-2 infection. Despite challenges such as limited access to COVID-19 patient tissue samples, the absence of rodent models that closely resemble SARS-CoV-2 infection and ethical concerns in clinical and basic research. Numerous valuable genomic data from COVID-19 patients are publicly available. For example, databases such as the National Center for Biotechnology Information (NCBI) provide a huge trove of valuable genomic data from COVID-19 patients. However, these data have not been utilized effectively. We utilized NCBI COVID-19 data sets and leveraged the curated findings in the QKB to examine the impact of alcohol consumption on COVID-19-associated pain. Using bioinformatic tools, the present study navigated possible interactions among alcohol consumption, SARS-CoV-2 infection, and pain biological systems. In our network meta-analysis, we revealed that alcohol exhibits analgesic properties. However, the combination of alcohol and SARS-CoV-2 infection was found to increase pain levels. In-silico analysis of post-mortem brain tissue samples of the PFC and amygdala of COVID-19 patients revealed that DEGs triggered by SARS-CoV-2 infection increase the immune responses of cells as well as progressive neuromuscular disease, progressive motor neuron disease, progressive neuropathy, and neuromuscular disease with neuropathy. Furthermore, simulation studies on the impact of EtOH exposure on DEGs from the PFC and amygdala of COVID-19 patients revealed that EtOH exposure leads to increased discomfort, neuropathic pain, and inflammatory pain.

According to the reports from NIAAA, monthly per capita sales of alcoholic beverages, including spirits and wine, increased in the United States during the COVID-19 lockdown period.^39^ Retail alcohol sales increased by 34% during the early pandemic throughout all geographic and demographic categories.^40^ The surge in sales was mainly driven by increased consumption due to coronavirus stay-at-home lockdowns, stockpiling, and a shift in customers' buying habits to bulk buying.^41^ Alcohol misuse or AUD in COVID-19 patients results in exaggerated SARS-CoV-2 infection-induced pathology.^17,18^ Increased incidence of pain reported in post-COVID-19 patients can also be influenced by alcohol misuse as it has a pronounced effect on pain processing and pathological development.

In the present study, we demonstrated that alcohol’s analgesic properties have been reported in numerous empirical studies. To determine the potential effects of alcohol on downstream molecules, we utilized the curated information in the QKB and IPA tools including “Connect” to generate a network map between EtOH and pain. The “MAP” tool was used to simulate enhanced EtOH exposure to mimic alcohol misuse. EtOH simulations showed a direct inhibitory effect on pain. In addition, EtOH simulation mediated the activation of NGFR, EHMT2, and IL10 and inhibition of TRPM8, resulting in an overall concurrent inhibition of pain or analgesic properties (Figure 1). The association of these molecules with alcohol misuse and discomfort has been reported in the literature. With the bioinformatics tools, we are able to construct a network map between EtOH and pain via the commonly shared molecules. NGFR protein was found to decrease inflammatory pain in mice exhibiting collagen-induced arthritis.^42^ The knocking out of the transcription factor EHMT2 in the central nucleus of the amygdala (CeA) increases pain sensitivity in a mouse model of inflammatory pain.^43^ EHMT2 is also involved in long noncoding RNAs (IncRNA that are highly expressed in dorsal root ganglion neurons (DS-lncRNA), mediating analgesia in nerve injury-induced hypersensitivity.^44^ The activation of the anti-inflammatory molecule, IL10, promotes analgesia by suppressing the production of inflammatory mediators.^45^ TRPM8 is critical in mediating thermal pain, specifically cold and nociceptive stimuli.^46^ Alcohol-mediated inhibition of TRPM8 also contributes to alcohol’s alteration of the pain.^47^

Our analysis found 134 commonly shared molecules between EtOH and SARS-CoV-2 The MAP simulation of enhanced EtOH exposure increased the discomfort mediated via the overlapping molecules between EtOH and SARS-CoV-2 infection. This finding suggests that common targets of EtOH and SARS-CoV-2 infection mediate pain synergistically. Among the overlapping molecules, IPA analysis revealed that, in the context of COVID-19, EtOH-mediated activation of PTGS2, IL6, TNF, and cytokines as well as inhibition of SIRT1 and ascorbic acid are critically involved in EtOH-induced pain. The association of the remaining molecules are minimal or inconsistent for IPA to predict their downstream effects (Figure 2B). One of the common pathological hallmarks of SARS-CoV2 infection and chronic alcohol consumption is that they both induce a cytokine storm. Consistent with our IPA findings, previous reports suggest that both alcohol consumption and SARS-CoV2 infection activate PTGS2, which encodes cyclooxygenase and produces pro-inflammatory prostaglandins.^48−51^ These prostaglandins increase peripheral and central sensitization and eventually lead to chronic pain conditions.^52^ Alcohol-triggered activation of cytokines, IL6, and TNF is associated with the development of all types of pain including neuropathic and inflammatory pain.^53−55^

**Figure 5:**
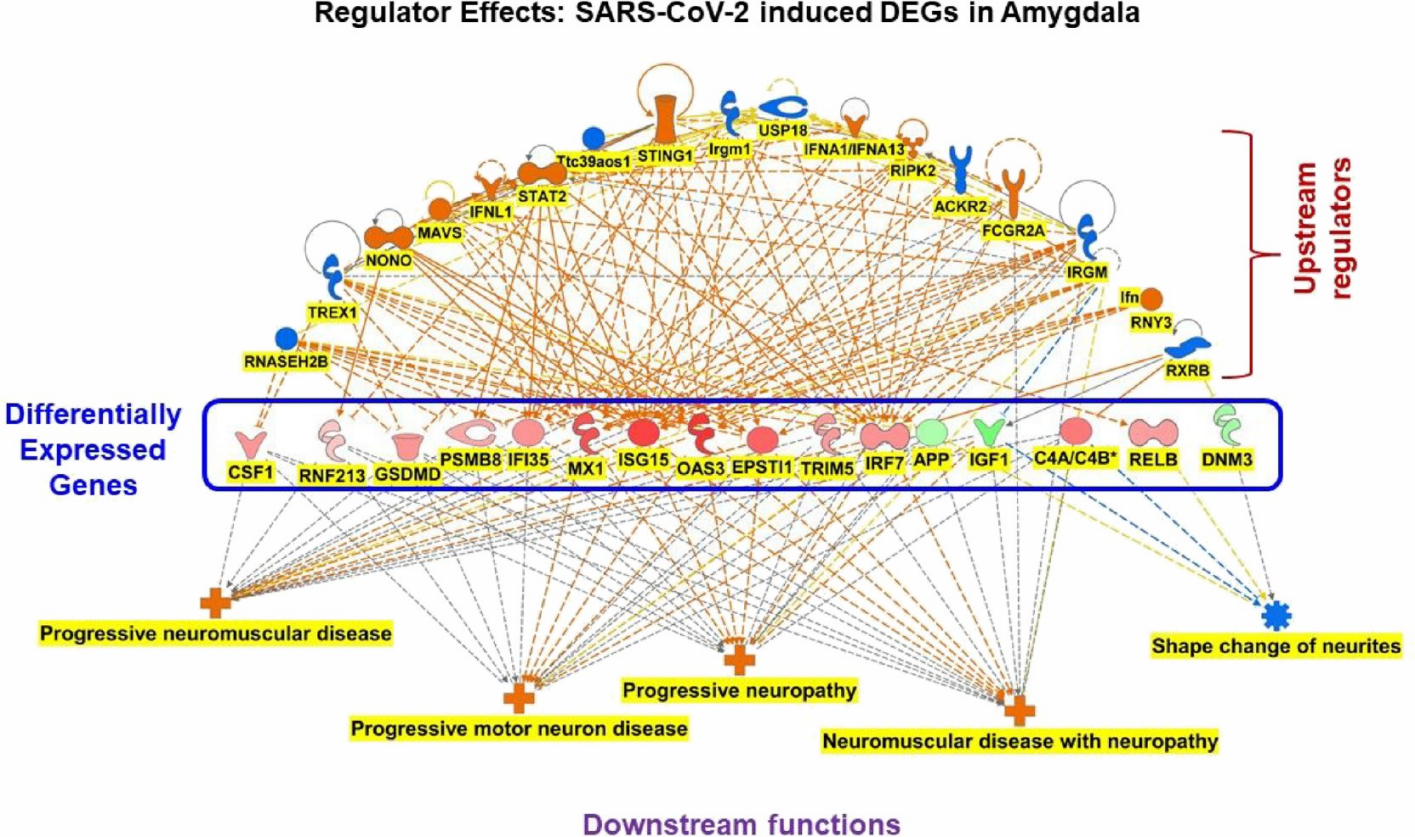
SARS-CoV-2 infection triggers upstream regulators, which results in the activation of the DEGs including CSF1, RNF213, SMD, PSM8, IF135, MX1, ISG15, OAS3, EPST1, TRIM5, IRF7, and RELB, and inhibition APP, IGF1, and DNM3, these activation and inhibition of the DEGs modulates progressive neuromuscular disease, and motor neuron disease, progressive neuropathy, and neuromuscular disease with neuropathy in the amygdala of the COVID-19 patients.

Our previous study was the first to demonstrate the brain targets of alcohol exposure. Using Fos immunoreactivity, we identified six brain regions including the bed nucleus of the stria terminalis (BNST), paraventricular hypothalamic nucleus (PVN), CeA, Edinger-Westphal nucleus (EW), locus coeruleus nucleus (LC), and parabrachial nucleus (PB) that showed the neuronal activation following alcohol exposure.^56^ Later studies revealed common neural substrates including PFC, nucleus accumbens (NAC), central amygdala (CeA), ventral tegmental area (VTA), and thalamus (THA), which are involved in mediating pain, alcohol intoxication, and dependence.^25−27^ In the present study, we accessed transcriptomic data derived from post-mortem brain tissue samples of COVID-19 patients available in the Gene Expression Omnibus. The available samples included data from the PFC and amygdala of the brain. IPA analysis of the DEGs from the PFC and amygdala revealed the signaling pathways and upstream regulators, including the transcription factors, that are associated with the observed DEGs in response to SARS-CoV-2 infection. IPA’s “regulator effect analysis” identified the potential functions or pathways controlled by the upstream regulators and downstream functions of the DEGs. IPA computes a consistency score with the highest score predicting a more accurate prediction of regulator effect. In the PFC of COVID-19 patients, the top upstream regulator IFNAR family regulates the activation of inflammatory responses and immune responses of the cells (Figure 4). Upstream regulators in the amygdala regulate the activation of progressive neuropathy and neuromuscular diseases with and without neuropathy (Figure 5). These findings are consistent with the previous findings from COVID-19 patients. Similarly, SARS-CoV-2 infection triggers neuroinflammation, leading to gliosis and injury in the PFC of COVID-19 patients.^57^ Findings from the amygdala are consistent with previous reports indicating COVID-19-mediated nociceptor excitability and neuropathic pain.^58^ The Increased production of IFN following SARS-CoV-2 infection plays a vital role in mediating cytokine storm, which is characterized by the release pro-inflammatory cytokines. This activation of pro-inflammatory cytokine cascades can further lead to neurogenic inflammation and nociceptor hypersensitivity and contribute to pathological conditions like inflammatory and neuropathic pain.^58^ Our IPA analysis also identified IFN receptor activation, particularly IFN, IFNL1, and IFNAR as the top upstream regulators in the brain regions, which mediate inflammation and neuropathic conditions (Figures 4 and 5). These findings suggest that SARS-CoV-2 infection can trigger pain signaling cascades in the brain.

To study whether alcohol exposure can exaggerate the pain induced by the SARS-CoV-2 virus, we performed an EtOH exposure simulation to examine the impact on the DEGs from the PFC and amygdala of COVID-19 patients. In the PFC, there were 548 DEGs commonly shared with EtOH, and the simulation exposure resulted in the activation of discomfort and neuropathic pain, but not inflammatory pain. Activation of neuropathic pain was mainly mediated via the EtOH-induced activation of TLR4. TLR4 activation has a pivotal role in mediating neuropathic pain by activating glial cells and sensory neurons which further influence nociceptive processing and pain.^59^ In the amygdala, only 39 DEGs overlapped with EtOH, and the simulation of EtOH activation resulted in discomfort and inflammatory pain (Figure 7). Discomfort is mainly mediated by the activation of potassium inwardly rectifying channel subfamily J member 6 (KCNJ6). KCNJ6 is associated with pain-related phenotypes and influences morphine’s analgesic properties in treating postoperative pain.^60^ It is important to note that the absence of observed effects of the biological molecules of interest in any of the pain nodes (discomfort, neuropathic pain, and inflammatory pain) may be due to the unavailability of data in the QKB. However, it opens future directions to study these targets in the context of alcohol, COVID-19, and pain. The number of DEGs is smaller in the amygdala compared to the PFC. This suggests that specific brain regions change in response to SARS-CoV-2 infection. In addition, the virus invades the brain via mechanisms of altered BBB and S1 spike protein,^61,62^ while the degrees of invasion in the brain parenchyma are yet to be identified.

**Figure 6:**
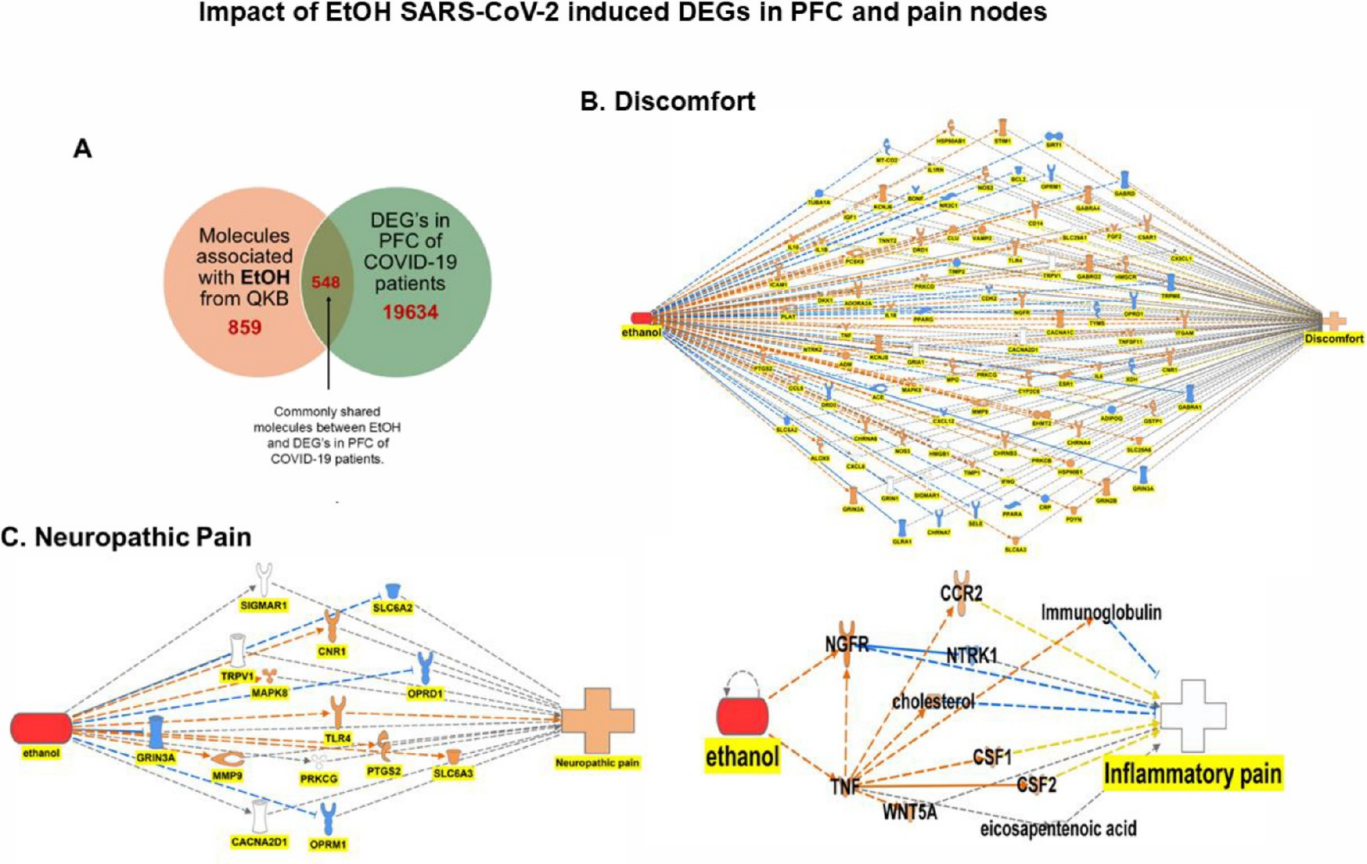
Impact of EtOH exposure on various pain nodes in IPA. (A) EtOH and SARS-CoV-2 infection induced DEGs commonly shared molecules in the PFC. Enhancement of EtOH in simulation results in increased (B) discomfort, (C) neuropathic pain, and (D) inflammatory pain remained unchanged.

**Figure 7:**
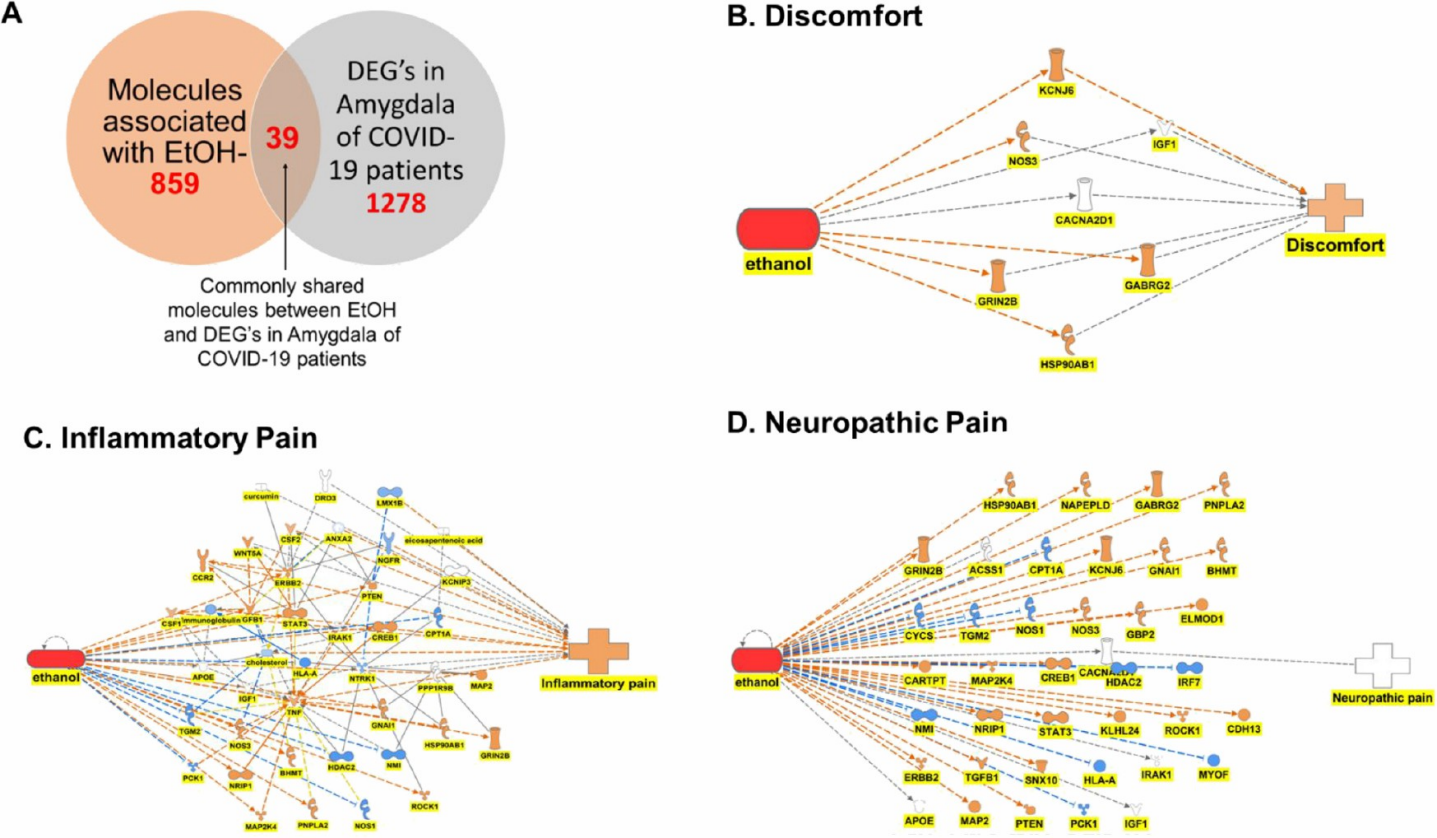
Impact of EtOH exposure on various pain nodes in IPA. (A) EtOH and SARS-CoV-2 infection induced DEGs commonly shared molecules (A) commonly in the amygdala. Enhancement of EtOH in simulation results in an increase in (B) discomfort and (C) inflammatory pain but (D) neuropathic pain remained unchanged.

Like other experimental methods, in silico analysis also has certain limitations. The main challenge we faced while curating the present data was the limited availability of COVID-19 data, as it is a relatively recent pandemic. IPA relies on the curated information available in QKB. Among the DEGs identified from the PFC and amygdala of COVID-19 patients, only a few overlapped with neuropathic pain and inflammatory pain. This limitation is due to the scarcity of existing data in the literature linking the association of COVID-19 and neuropathic and inflammatory pain. Currently, only epidemiological findings exist in the literature that report chronic pain among COVID-19 patients. Due to the lack of molecular mechanistic studies on chronic pain in COVID-19 patients, our EtOH simulation results depicted in Figures 6D and 7D did not show any changes in the neuropathic and inflammatory pain nodes following alcohol exposure. Using data-driven approaches, the present study revealed empirical evidence of the role of alcohol modulation of chronic pain in COVID-19 patients.

## 5 Conclusions

The present study aimed to identify the influence of alcohol consumption and COVID-19 on pain. Using publicly available gene expression data sets of DEGs in the PFC and amygdala of COVID-19 patients, we employed a bioinformatics application, QIAGEN ingenuity pathway analysis (IPA), to identify the key signaling pathways, upstream regulators, and biological functions in these brain areas known to play a role in pain. We found that while alcohol consumption directly inhibits pain, the presence of COVID-19 exaggerates impaired cytokine signaling, neuroinflammation, and neuropathic pain signaling in the CNS providing novel insights into the signaling pathways associated with chronic pain in the COVID-19 patients.

## Supporting Information Available

8722 significant DEGs in the DEG analysis of the PFC of COVID-19 patients (XLS)
1278 significant DEGs in the amygdala of COVID-19 patients (XLS)

## Abbreviations

ACE: angiotensin-converting enzyme
ACKR2: atypical chemokine receptor 2
ADM: adrenomedullin
ADORA2A: adenosine A2A receptor
ALOX5: arachidonate 5-lipoxygenase
APP: amyloid precursor protein
AUD: alcohol use disorder
BBB: blood–brain barrier
BDNF: brain-derived neurotrophic factor
C5AR1: C5a receptor 1
CACNA1C: calcium voltage-gated channel subunit alpha1 C
CACNA2D1: calcium voltage-gated channel subunit alpha2 delta 1
CAMKK2: calcium/calmodulin dependent protein kinase kinase 2
CASP1: caspase 1
CCL2: C–C motif chemokine ligand 2 (MCP-1)
CCL5: C–C motif chemokine ligand 5
CD14: cluster of differentiation 14
CD74: CD74 molecule, major histocompatibility complex, class II invariant chain
CD86: CD86 molecule (also known as B7.2)
CeA: central amygdala
CHRNA4: cholinergic receptor nicotinic alpha 4 subunit
CHRNA6: cholinergic receptor nicotinic alpha 6 subunit
CHRNA7: cholinergic receptor nicotinic alpha 7 subunit
CHRNB3: cholinergic receptor nicotinic beta 3 subunit
CLU: clusterin
CNS: central nervous system
COVID-19: coronavirus disease 2019
CRP: C-reactive protein
CSF1: colony stimulating factor 1 (macrophage colony-stimulating factor)
CXCL10: C-X-C motif chemokine ligand 10 (IP-10)
CYP2B6: cytochrome P450 2B6
DEG: differentially expressed genes
DNM3: dynamin 3
DOCK8: dedicator of cytokinesis 8
DRD1: dopamine receptor D1
DRD2: dopamine receptor D2
EHMT2: euchromatic histone lysine methyltransferase 2
EPSTI1: epidermal growth factor stimulated gene 1
ESR1: estrogen receptor 1
EtOH: ethanol (alcohol)
FCER1G: Fc epsilon receptor 1 subunit gamma
FCGR2A: Fc gamma receptor IIa
GABA-A: gamma-aminobutyric acid type A
GABRG2: gamma-aminobutyric acid type A receptor gamma2 subunit
GEO: Gene Expression Omnibus
GRIN2B: glutamate ionotropic receptor NMDA type subunit 2B
GRIN3A: glutamate ionotropic receptor NMDA type subunit 3A
GRN: granulin
GSDMD: gasdermin D
HMGB1: high mobility group box 1
HMGCR: 3-hydroxy-3-methylglutaryl-CoA reductase
HSP90AB1: heat shock protein 90 alpha family class B member 1
ICAM1: intercellular adhesion molecule 1
IFI35: interferon induced protein 35
IFIH1: interferon induced with helicase C domain 1
IFNA1: interferon alpha 1
IFNA10: interferon alpha 10
IFNA14: interferon alpha 14
IFNA16: interferon alpha 16
IFNA2: interferon alpha 2
IFNA21: interferon alpha 21
IFNA5: interferon alpha 5
IFNA6: interferon alpha 6
IFNA7: interferon alpha 7
IFNA8: interferon alpha 8
IFNAR: interferon alpha receptor
IFNAR2: interferon alpha receptor subunit 2
IFNG: interferon gamma
IFNGR1: interferon gamma receptor 1
IFNL1: interferon lambda 1
IFNL4: interferon lambda 4
IGF1: insulin-like growth factor 1
IL: interleukin
IL1: interleukin 1
IL10: interleukin 10
IL18: interleukin 18
IL1B: interleukin 1 beta
IL2: interleukin 2
IL6: interleukin 6
IPA: ingenuity pathway analysis
IRF1: interferon regulatory factor 1
IRF3: interferon regulatory factor 3
IRF7: interferon regulatory factor 7
IRF9: interferon regulatory factor 9
IRGM: immunoresponse gene M
ISG15: interferon stimulated gene 15
ITCH: itchy E3 ubiquitin protein ligase
KCNJ5: potassium inwardly rectifying channel subfamily J member 5
KCNJ6: potassium inwardly rectifying channel subfamily J member 6
LTBP1: latent transforming growth factor beta binding protein 1
MAP: molecular activity predictor
MAPK1: mitogen-activated protein kinase 1
MAPK8: mitogen-activated protein kinase 8
MAVS: mitochondrial antiviral signaling protein
MD: Maryland
miRNA: microRNA
MMP9: matrix metallopeptidase 9
MVAS: myocardial vascularization
MX1: MX dynamin like GTPase 1
MYD88: myeloid differentiation primary response gene 88
NAc: nucleus accumbens
NCBI: national center for biotechnology information
NFATC2: nuclear factor of activated T-cells cytoplasmic 2
NGFR: nerve growth factor receptor
NLRP3: NOD-like receptor family pyrin domain containing 3
NMDA Receptor: *N*-methyl-d-aspartate receptor
NOD1: nucleotide binding oligomerization domain 1
NOD2: nucleotide binding oligomerization domain 2
NONO: non-POU domain containing octamer-binding protein
NOS3: nitric oxide synthase 3 (Endothelial)
NR3C1: nuclear receptor subfamily 3 group C member 1
NRTN: neurturin
NVAS: neurovasculature
OAS3: 2′-5′ oligoadenylate synthetase 3
PARP9: poly(ADP-ribose) polymerase family member 9
PCSK9: proprotein convertase subtilisin/kexin type 9
PDCD1LG2: programmed cell death 1 ligand 2
PDYN: prodynorphin
PFC: prefrontal cortex
PNPT1: polyribonucleotide nucleotidyltransferase 1
PPARA: peroxisome proliferator-activated receptor alpha
PPARG: peroxisome proliferator-activated receptor gamma
PRKCD: protein kinase C delta
PSMB8: proteasome subunit beta 8
PTGS2: prostaglandin-endoperoxide synthase 2
PTGS2: prostaglandin-endoperoxide synthase 2 (COX-2)
QKB: QIAGEN Knowledge Base
qPCR: quantitative polymerase chain reaction
REL B: REL B proto-oncogene, NF-KB subunit
REST: RE1-silencing transcription factor
RIGI: retinoic acid-inducible gene I
RIPK2: receptor interacting serine/threonine kinase 2
RNASEH2B: ribonuclease H2 subunit B
RNY3: RNA Y-Box binding protein 3
RXRB: retinoid X receptor beta
S1: spike protein (from SARS-CoV-2)
SAMSN1: SAM and SH3 domain containing 1
SARS-CoV-2: severe acute respiratory syndrome coronavirus 2
SASH1: SAM and SH3 domain containing 1
SIRT1: sirtuin 1
SLC25A6: solute carrier family 25 member 6
SMD: Severe Motor Deficit
STAT1: signal transducer and activator of transcription 1
STAT2: signal transducer and activator of transcription 2
STING1: stimulator of interferon genes 1
TAP1: antigen peptide transporter 1
TIMP1: tissue inhibitor of metalloproteinases 1
TLR2: toll-like receptor 2
TLR3: toll-like receptor 3
TLR4: toll-like receptor 4
TNF: tumor necrosis factor
TNFSF11: tumor necrosis factor superfamily member 11
TREX1: three prime repair exonuclease 1
TRIM21: tripartite motif containing 21
TRIM24: tripartite motif containing 24
TRIM5: tripartite motif containing 5
TRPM8: transient receptor potential cation channel subfamily M member 8
TRPM8: transient receptor potential melastatin 8
TTC39AOS1: tetratricopeptide repeat domain 39A overlapping with shortened exon 1
TUBA1A: tubulin alpha 1A
UBE2L6: ubiquitin conjugating enzyme E2 L6
VAMP2: vesicle-associated membrane protein 2
VCAM1: Vascular Cell Adhesion Molecule 1
VEGFA: vascular endothelial growth factor A
VTA: ventral tegmental area
WHO: World Health Organization
